# Repurposing of Biologic and Targeted Synthetic Anti-Rheumatic Drugs in COVID-19 and Hyper-Inflammation: A Comprehensive Review of Available and Emerging Evidence at the Peak of the Pandemic

**DOI:** 10.3389/fphar.2020.598308

**Published:** 2020-12-18

**Authors:** Giulio Cavalli, Nicola Farina, Corrado Campochiaro, Giacomo De Luca, Emanuel Della-Torre, Alessandro Tomelleri, Lorenzo Dagna

**Affiliations:** ^1^ Unit of Immunology, Rheumatology, Allergy and Rare Diseases (UnIRAR), IRCCS San Raffaele Hospital, Milan, Italy; ^2^ Vita-Salute San Raffaele University, Milan, Italy

**Keywords:** Coronavirus disease 2019, severe acute respiratory syndrome coronavirus 2, disease modifying anti-rheumatic drug, DMARDs (biologic), cytokine, immunesuppressants, JAK inhibitors

## Abstract

Coronavirus disease 2019 (COVID-19) is a condition caused by the severe acute respiratory syndrome coronavirus 2 (SARS-CoV-2). Severe cases of COVID-19 result in acute respiratory distress syndrome and death. A detrimental, hyper-inflammatory immune response with excess release of cytokines is the main driver of disease development and of tissue damage in these patients. Thus, repurposing of biologic agents and other pharmacological inhibitors of cytokines used for the treatment of various inflammatory conditions emerged as a logical therapeutic strategy to quench inflammation and improve the clinical outcome of COVID-19 patients. Evaluated agents include the interleukin one receptor blocker anakinra, monoclonal antibodies inhibiting IL-6 tocilizumab and sarilumab, monoclonal antibodies inhibiting granulocyte-monocyte colony stimulating factor and tumor necrosis factor, and Janus kinase inhibitors. In this review, we discuss the efficacy and safety of these therapeutic options based on direct personal experience and on published evidence from observational studies and randomized clinical trials.

## Introduction

**FIGURE 1:**
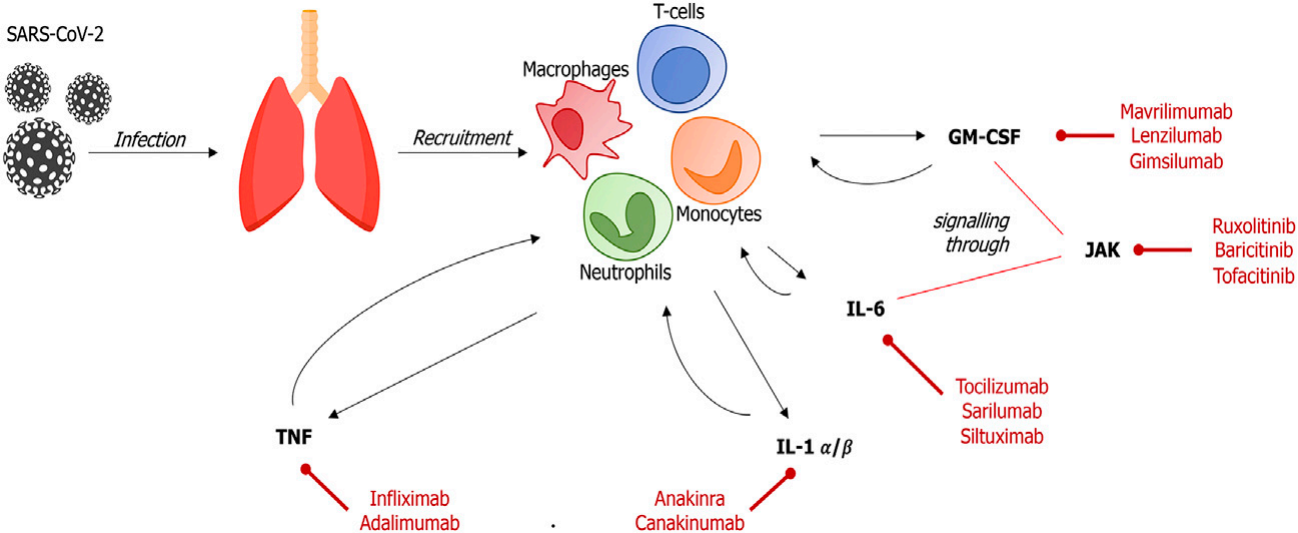
Main pathways and treatment targets in SARS-CoV-2–induced immune response In the early stage of SARS-CoV-2 infection, infected cells and resident macrophages release signaling molecules that recruit host immune cells into the alveolar space. These cells, mainly neutrophils, T-lymphocytes and monocytes, produce and release high levels of inflammatory cytokines, leading to an uncontrolled inflammatory response (GM-CSM, granulocyte-monocyte colony-stimulating factor; IL, interleukin; JAK, Janus kinase; TNF, tumor necrosis factor).

The pathogenesis of severe Coronavirus disease 2019 (COVID-19) involves an excessive, maladaptive host inflammatory response to the causative virus SARS-CoV-2 (Mehta et al., 2020; Ruan et al., 2020) (Figure 1). Individual predisposition to the development of excessive or inappropriate immune responses is traditionally attributed to genetic variation in the genes encoding the human leukocyte antigen (HLA) (Cavalli et al., 2016a; Hayashi et al., 2016; Klück et al., 2020). Conversely, the detrimental immune response developing in a subgroup of COVID-19 patients is mediated by the innate immune system, and is characterized by marked increases in systemic cytokines, and is paralleled by elevations in inflammatory biomarkers, such as C-reactive protein (CRP) and ferritin (Ciceri et al., 2020; Campochiaro et al., 2020a; Colafrancesco et al., 2020b; Fominskiy et al., 2020; Mehta et al., 2020; Zangrillo et al., 2020). A similar biochemical pattern was observed in severe patients affected by pneumonia caused by previous coronaviruses SARS-CoV and MERS-CoV (Wong et al., 2004; Mahallawi et al., 2018).

Severe forms of COVID-19 pneumonia feature elevations in circulating levels of interleukin (IL) 1, IL-6, granulocyte-macrophage colony-stimulating factor (GM-CSF) and tumor necrosis factor α (TNF) (Ragab et al., 2020; Rovere-Querini et al., 2020; Tang N. et al., 2020)*.* Conversely, decreased levels of interferon I are associated with disease severity (Bastard et al., 2020; Hadjadj et al., 2020; Zhang et al., 2020).

The hyper-inflammatory response turning COVID-19 into a life-threatening disease shares conceptual and molecular resemblance with the cytokine storm developing during the macrophage activation syndrome, or with the cytokine release syndrome following chimeric antigen receptor T (CAR-T)-cell therapy (De Luca et al., 2020). Thus, pharmacological inhibition of several pro-inflammatory cytokines, along with a broader therapeutic molecular blockade (eg inhibition of Janus Kinases [JAK]), has been extensively explored during the COVID-19 pandemic (Ciceri et al., 2020; Campochiaro et al., 2020a; Campochiaro et al., 2020b; Della-Torre et al., 2020; Fominskiy et al., 2020; Mehta et al., 2020; Zangrillo et al., 2020). In this review, we discuss the biologic rationale for repurposing of available anti-cytokine therapies, as well as the available evidence on the effectiveness of different pharmacological blockers of inflammatory mediators in COVID-19 (Table 1).

**TABLE 1:** Main published observational studies and randomized trials of biologic and targeted synthetic drugs for the treatment of SARS-CoV-2–induced hyperinflammation.

Agent	Ref	Study information	Sample size	Study population	Setting	Main results
Interleukin-1						
Anakinra	Cavalli et al. (2020a)	Single-centre, open-label	29 treated	Respiratory failure, hyperinflammation	Outside ICU	Improved respiratory function, improved survival
			16 controls			
Anakinra	Huet et al. (2020)	Single-centre, open-label	52 treateds	Respiratory failure	Outside ICU	Reduced ICU admission, improved survival
			44 control			
Anakinra	Navarro‐Millán et al. (2020)	Single-centre, case-series	11 treated	Respiratory failure, hyperinflammation	Outside ICU	7 patients not required invasive mechanical ventilation (early-treated)
canakinumab	Ucciferri et al. (2020)	Single-centre, case-series	10 treated	Respiratory failure, hyperinflammation	Outside ICU	All patients discharged
Interleukin-6						
Tocilizumab	Campochiaro et al. (2020a)	Single-centre, open-label	52 treated	Respiratory failure, hyperinflammation	Outside ICU	No differences in clinical improvement and survival
			44 controls			
Tocilizumab	Capra et al. (2020)	Single-centre, open-label	62 treated	Respiratory failure	Outside ICU	Improved respiratory function, improved survival
			23 controls			
Tocilizumab	Morena et al. (2020)	Single-centre, case series	51 treated	Respiratory failure, hyperinflammation	ICU and non-ICU	31 patients were discharged, 17 had a worsening of the clinical status, 14 died
Tocilizumab	Guaraldi et al. (2020)	Multicentre, open-label	179 treated	Respiratory failure	Outside ICU	Reduced ICU admission or death
			365 controls			
Tocilizumab	Biran et al. (2020)	Multicentre, open-label	210 treated	ARDS with mechanical support	ICU	Improved survival
			420 controls			
Tocilizumab	Stone et al. (2020)	Multicenter RCT	161 treated	Respiratory failure, hyperinflammation	Outside ICU	No differences in clinical improvement and survival
			82 controls			
Tocilizumab	Hermine et al. (2020)	Multicenter RCT	64 treated 67 controls	Respiratory failure, hyperinflammation	Outside ICU	Reduced mechanical ventilation and death rate at 14 days; no differences in survival at 28 days
Tocilizumab/sarilumab	Sinha et al. (2020)	Single-centre, case series	255 treated	Respiratory failure, hyperinflammation	Outside ICU	Mortality of severe patients was comparable to the overall COVID-19-related mortality in the local area
sarilumab	Benucci et al. (2020)	Single-centre, case series	8 treated	Respiratory failure	Outside ICU	7 patients discharged within 14 days, 1 patient died
sarilumab	Della-Torre et al. (2020)	Single-centre, open-label	28 treated	Respiratory failure, hyperinflammation	Outside ICU	No differences in clinical improvement and survival
			28 controls			
GM-CSF						
Mavrilimumab	De Luca et al. (2020)	Single-centre, open-label	13 treated	Respiratory failure, hyperinflammation	Outside ICU	Greater and earlier improvement of clinical outcomes
			26 controls			
Tumor necrosis factor						
Infliximab	Stallmach et al. (2020)	Single-centre, open-label	7 treated	Respiratory failure, hyperinflammation	Outside ICU	Clinical improvement in 6 patients
			17 controls			
Janus kinases						
Ruxolitinib	Cao et al. (2020)	Multicenter RCT	20 treated	Respiratory failure	Outside ICU	Faster clinical recovery; chest CT improvement
			21 controls			
Baricitinib	Cantini et al. (2020)	Multicentre, open-label	113 treated	Respiratory failure	Outside ICU	Improved respiratory function, reduced ICU admission, increased discharge rate
			78 controls			

### Interleukin one

IL-1 is the prototypical pro-inflammatory cytokine. Two different gene products, IL-1α and IL-1β, can activate the IL-1 receptor. IL-1α is constitutively present as an active molecule in all mesenchymal and epithelial tissues; it is released upon cell death, and acts as an alarmin inducing local inflammation (Rider et al., 2017). IL-1β is not detectable in healthy tissues and is secreted in the extracellular space during inflammation (Dinarello, 2009; Cavalli and Cenci, 2020; Klück et al., 2020). Both IL-1α and IL-1β bind the same receptor and induce several pro-inflammatory effects (Dinarello, 2009; Rider et al., 2017; Ragab et al., 2020; Vecchié et al., 2020).

Although mechanistic insight into the host inflammatory response to COVID-19 is still limited, it is likely that both IL-1α and IL-1β play a central role in the development of the exuberant, maladaptive inflammatory response leading to life-threatening states in some patients (Ben Salem, 2017; Mehta et al., 2020; Ruan et al., 2020). Specifically, damaged epithelial and endothelial tissues release IL-1α in the lung, whereas infiltrating myeloid cells produce abundant IL-1β (Dinarello, 2011).

The main physiologic mechanism preventing runaway IL-1-mediated inflammation is the IL-1 receptor antagonist (IL-1Ra) (Gabay et al., 1997; Arena et al., 1998; Park et al., 2001). Anakinra is a recombinant form of IL-1Ra and the first-in-class IL-1 inhibitor drug (Cavalli and Dinarello, 2015; Cavalli and Dinarello, 2018). It is used for the treating rheumatoid arthritis, autoinflammatory disorder and multiple diseases characterized by excess cytokine production, including critical disease states (Abbate et al., 2015; Cavalli et al., 2015a; Cavalli et al., 2017; Tomelleri et al., 2018; Campochiaro et al., 2019). Notable therapeutic applications include adult-onset Still’s disease (Cavalli et al., 2015a; Campochiaro et al., 2020b; Cavalli et al., 2020b) and macrophage activation syndrome (Grom et al., 2016; Ravelli et al., 2016; Eloseily et al., 2020), both conditions sharing similarities with COVID-19 and hyper-inflammation. In addition, re-analysis of a trial of anakinra in sepsis confirmed clinical benefits in patients with features of hyper-inflammation (Shakoory et al., 2016). A good safety profile and a short half-life of 3 h, which ensures rapid clearance from the circulation, contributes to making anakinra a suitable treatment for critically ill patients (Cavalli and Dinarello, 2015).

Based on extensively documented safety and effectiveness in quenching hyper-inflammation in multiple diseases, including cardiopulmonary insufficiencies (Cavalli et al., 2015b; Cavalli et al., 2016a; De Luca et al., 2018; Sala et al., 2020), anakinra was among the first cytokine-blocking agents evaluated for the treatment of COVID-19, as documented by multiple reports (Aouba et al., 2020; Dimopoulos et al., 2020; Pontali et al., 2020). In the first cohort study by Cavalli et al., administration of high-dose intravenous anakinra quenched hyper-inflammation and improved respiratory function in 29 severe patients with COVID-19 ARDS receiving non-invasive ventilation (NIV) (Cavalli et al., 2020). This amounted to improved survival in treated patients compared to concomitantly hospitalized patients who did not receive anakinra. Subsequent cohort studies by Huet et al. and Navarro-Millàn independently confirmed these findings in different disease severity stages (Huet et al., 2020; Navarro‐Millán et al., 2020). In addition, the effectiveness of anakinra has been reported in different case series (Aouba et al., 2020; Cavalli et al., 2020; Dimopoulos et al., 2020; Huet et al., 2020; Navarro‐Millán et al., 2020; Pontali et al., 2020).

The positive findings of these studies are to be interpreted with caution in view of possible biases (i.e. single-center study bias, small study bias), as well as the limited number and uncontrolled nature of the investigations. Furthermore, the dosage regimens for anakinra varied across studies, ranging from high-dose intravenous administration in the study by Cavalli et al., to relatively low dose subcutaneous administration in the study by Huet et al. (Cavalli et al., 2020a; Huet et al., 2020). The timing of administration also differed between studies due to practical reasons, although all investigators shared a conceptual attitude toward the earliest possible administration. For these limitations, no indication on which anakinra regimen is most suitable for COVID-19 can be extrapolated from these studies. However, given the safety of anakinra even at high doses, early and aggressive treatment (i.e. 10 mg/kg/day intravenously) is probably advisable, in line with current management of autoinflammatory diseases and macrophage activation syndrome (Grom et al., 2016; Ravelli et al., 2016; Eloseily et al., 2020; Vitale et al., 2020). Clinical trials of anakinra in COVID-19 are ongoing (i.e. NCT04443881 among others). If ever available, controlled evidence from these investigations will supersede currently available observational evidence.

Besides anakinra, another IL-1 antagonist was evaluated in COVID-19, that is, the anti-IL-1β monoclonal antibody canakinumab. Canakinumab is used for the treatment of adult autoinflammatory conditions such as Still’s disease (Colafrancesco et al., 2017; Cavalli et al., 2019). It does not block IL-1α. Experience with canakinumab in COVID-19 is limited to a single, small case series reporting favorable responses (Ucciferri et al., 2020). Clinical trials of canakinumab are also ongoing (i.e. NCT04362813).

### Interleukin six

IL-6 is a pleiotropic cytokine produced by virtually every immune cell types, which acts by engaging its receptor (IL-6R) on target cells (De Benedetti et al., 2012). IL-6 is involved in physiological hematopoiesis and response to pathogens but excessive production is associated with disorders that resemble severe COVID-19 manifestations, such as the hemophagocytic lymphohistiocytosis, and the cytokine release syndrome induced by CAR-T-cell (Henter et al., 1991; Lee et al., 2014; Tanaka et al., 2016; Chen et al., 2019; Aziz et al., 2020; Cavalli et al., 2020; Kaur et al., 2020; Ruan et al., 2020). Stemming from preliminary evidence of increased pro-inflammatory cytokines in sera and bronchoalveolar lavage of patients with COVID-19 pneumonia, IL-6 attracted remarkable attention as a possible player in the pathogenesis of SARS-CoV-2 infection and in the hyper-inflammatory response that affects patients with severe disease (Blanco-Melo et al., 2020; Conti et al., 2020; Farina et al., 2020; Giamarellos-Bourboulis et al., 2020). Indeed, elevated serum levels of IL-6 were described to be associated to poorer outcomes, coagulopathy, and increased mortality in patients with COVID-19 (Chen et al., 2020).

Based on this evidence, several IL-6 inhibitory agents such as tocilizumab and sarilumab were repurposed in the setting of severe COVID-19. Tocilizumab, a monoclonal antibody against the IL-6R, was the first biologic agent to be largely evaluated in COVID-19 patients, also based on precipitous inclusion in the Chinese guidelines for the treatment of COVID-19 patients at the beginning of the pandemic (Di Giambenedetto et al., 2020). Tocilizumab is currently approved for the treatment of multiple inflammatory diseases (Berti et al., 2015; Stone et al., 2017; Le et al., 2018), and is used off-label to treat several inflammatory conditions (Berti et al., 2017). Tocilizumab is available in America, Asia, Europe and Oceania; however, it is not universally accessible as it has been approved for use only in few African countries (Akintayo et al., 2020). The first reported experience on tocilizumab in COVID-19 was described in a Chinese cohort of 15 patients. Tocilizumab was administered intravenously, at various dosages (from 80 to 480 mg), and five patients received more than one dose. These patients were followed-up for 7 days, and three of them died. This study showed preliminary encouraging results, but it was limited by the lack of a standardized therapeutic scheme, the absence of a control arm, and the short post-treatment follow-up. Moreover, eight patients were also concomitantly treated with steroid therapy making it hard to clearly investigate the role of anti-IL-6 blockade (Luo et al., 2020). Subsequent observational retrospective series of critically ill Chinese COVID-19 patients treated with tocilizumab also reported a decrease in CRP levels, mechanical ventilation risk and mortality rate (Xu et al., 2020). Similar findings were reported irrespective of the route of administration, either intravenous or subcutaneous (Sciascia et al., 2020).

Based on these pioneering observations from China and following the westbound spread of the pandemic, a series of Italian studies evaluated off-label use of tocilizumab in COVID-19 patients. Campochiaro and colleagues studied 65 patients with hyper-inflammation and observed a non-significant decrease in mortality at 28 days in 32 tocilizumab-treated patients (16%) compared to 33 patients treated with standard of care (33%); tocilizumab was administered at a dose of 400 mg (Campochiaro et al., 2020a). In a separate cohort, Capra and colleagues evaluated 85 severe COVID-19 patients and observed a mortality at 20 days of 3% in the 62 patients treated with tocilizumab (33 patients received 400 mg intravenously, two received 800 mg intravenously, and 27 received 324 mg subcutaneously) compared to 48% in the 23 patients treated with standard of care (Capra et al., 2020). Morena and colleagues observed clinical and bioumoral improvement in 51 patients with severe COVID-19 following tocilizumab infusion (two sequential infusions at the dosage of 400 mg) (Morena et al., 2020). Reported adverse events in these three studies did not differ between patients treated with tocilizumab or standard of care only: specifically, the Authors reported hepatic enzyme elevation in 15–29% of cases; neutropenia in 14–16% of cases; and bacterial or fungal infections in 13–27% of patients.

More recently, Guaraldi et al. reported the results of a large retrospective observational cohort study evaluating the efficacy of tocilizumab in the treatment of severe COVID-19 patients. They found no difference in need for mechanical ventilation between groups (16% of the standard of care group vs. 18% of the tocilizumab group, *p* = 0.41), but reported a statistically significant reduction in mortality in the tocilizumab group (7% vs. 20%, *p* < 0.001). At multivariate analysis tocilizumab was associated with a reduced risk of invasive mechanical ventilation or death (*p* = 0.020). However, an increased rate of secondary infection was observed in tocilizumab-treated patients (13% vs. 4%, *p* < 0.001) (Guaraldi et al., 2020).

In another study, Biran and colleagues analyzed 764 COVID-19 patients in the ICU, of whom 210 received tocilizumab. At multivariable analysis with propensity matching, tocilizumab was associated with a decreased hospital-related mortality (*p* = 0.004) (Biran et al., 2020).

Despite intrinsic limitations due to their retrospective nature, the absence of adequate controls, and the low statistical power, these and other promising experiences soon prompted initiation of randomized placebo-controlled trials aimed to evaluate the safety and efficacy of tocilizumab (NCT04377750, NCT04330638, NCT04322773) (Levi, 2020). Interim updates on the first randomized trial (COVACTA) investigating tocilizumab in severe COVID-19 pneumonia yielded disappointing results. At 4 weeks, there were no differences in clinical between patients receiving tocilizumab or placebo (*p* = 0.36). Also, there were no differences either in mortality rate, ventilator-free survival, and incidence of infections between the two groups (Roche, 2020). In a multicenter randomized trial involving 243 COVID-19 patients with signs of hyperinflammation, tocilizumab did not lead to a significant reduction of mortality or need for mechanical ventilation, nor it reduced the need for supplemental oxygen at 28 days (Stone et al., 2020). These findings were partially confirmed by another large RCT of COVID-19 patients (Hermine et al., 2020). In this trial, tocilizumab (administered at the dose of 8 mg/kg) led to a reduction in mechanical ventilation and death rate at 14 days; however, mortality at 28 days did not differ between treated patients and controls. Other parallel trials with tocilizumab in COVID-19 have been launched and results are expected by the end of the year (https://clinicaltrials.gov/; Campochiaro and Dagna, 2020).

Sarilumab is another anti IL-6R monoclonal antibody that was repurposed for the management of severe COVID-19 (https://www.accessdata.fda.gov/drugsatfda_docs/label/2017/761037s000lbl.pdf). Sarilumab shares the mechanism of action with tocilizumab, by blocking both the membrane bound and the soluble form of IL-6R (Burmester et al., 2017). In analogy with the pioneering experiences with tocilizumab, the first uncontrolled experiences with sarilumab also created positive expectations and inspired a series of randomized, double-blind, placebo-controlled phase II/III trials worldwide (NCT04357808, NCT04386239, NCT04324073, NCT04322773). Benucci et al., for instance, treated a small series of eight hospitalized COVID-19 patients with 400 mg intravenous sarilumab and reported clinical improvements in seven patients who were discharged before day 14 (Benucci et al., 2020). In a larger study from the epidemic New York City area in the United States, Sinha and colleagues administered either intravenous tocilizumab (400 mg) or sarilumab (200 mg) to 255 critical COVID-19 patients. The mortality rate of treated patients was comparable to the overall mortality in the local area, despite the notable severity of the study population (Sinha et al., 2020). However, in an observational prospective study on 56 Italian patients with severe COVID-19, sarilumab treatment did not result in incremental survival benefit at 28 days (Della-Torre et al., 2020). Additional evidence of limited efficacy of sarilumab in COVID-19 was provided by the early termination of a randomized trial led in the US. In this RCT sarilumab treatment was not associated with statistically significant differences in clinical outcomes. There was a favorable trend of clinical improvement and mortality in patients on mechanical ventilation, but also an unfavorable trend in non-mechanically ventilated subjects (Sanofi, 2020). Based on these results, the trial was stopped, and an originally planned extension trial evaluating higher doses of sarilumab (800 mg) did not take place. A separate trial evaluating the efficacy of sarilumab (administered at the dosage of 200 or 400 mg) in 420 critical patients also suggested a positive trend without reaching statistical significance (Regeneron Pharmaceuticals, Inc., 2020).

Finally, another IL-6 antagonist that was deemed of interest for severe COVID-19 patients is siltuximab, an FDA approved chimeric monoclonal antibody used for the management of neoplastic diseases such as metastatic renal cell cancer and Castleman’s disease (https://www.ema.europa.eu/en/medicines/human/EPAR/sylvant). Although there are no published experiences supporting the use of siltuximab in COVID-19 patients, similar effects to tocilizumab and sarilumab can be anticipated given the quasi-overlapping mechanism of action. A randomized trial evaluating the efficacy and safety of siltuximab (alone or in combination with anakinra) in hospitalized patients with severe COVID-19 (NCT04330638) is ongoing in Belgium, which will compare the efficacy of siltuximab to other anti-cytokine drugs (namely anakinra and tocilizumab) as well as to local standard of care.

Overall, available evidence from RCTs indicate that IL-6 inhibition is marginally or not effective for the treatment of COVID-19 (Hermine et al., 2020; Stone et al., 2020). In contrast, dexamethasone, a corticosteroid with broad anti-inflammatory properties significantly reduced mortality in a RCT of COVID-19 patients requiring supplemental oxygen or mechanical ventilation (Horby et al., 2020). IL-6 is a downstream, effector mediator of multiple inflammatory cascades. It is likely that in the massively inflamed lung of COVID-19, selective inhibition of IL-6 blocks but one of many mediators with redundant pro-inflammatory functions. This hypothesis also reconciles the negative findings of studies evaluating IL-6 inhibitors with the uncontrolled evidence suggesting that IL-1 inhibition might be effective for COVID-19: indeed, IL-1 is found more upstream in inflammatory cascades than IL-6. It is thereby likely that corticosteroids and IL-1 inhibition result in the inhibition of IL-6, as well as other mediators with a causative ole in the pathogenesis of COVID-19.

It should also be noted that observations of high circulating levels of IL-6 in COVID-19 patients can result in misled assumptions about the causal role of this cytokine in the pathogenesis of this disease. IL-6 levels are non-specifically elevated in systemic inflammation; in general, high circulating levels of any given cytokine do not indicate pathogenic causality, which is only demonstrated by the therapeutic effectiveness of selective cytokine inhibition.

### Granulocyte–Macrophage Colony-Stimulating Factor

GM-CSF is a cytokine with complex biologic activity, ranging from hematopoietic to pro-inflammatory effects (Shiomi and Usui, 2015; Crotti et al., 2019). Various cell types produce GM-CSF during inflammation, including macrophages, lymphocytes and tumor cells (Hamilton and GM-CSF, 2002; Shiomi and Usui, 2015; Xu et al., 2020)*.* GM-CSF activates several pro-inflammatory pathways and increases secretion of downstream mediators (Hamilton, 2019). Of note, GM-CSF can be placed upstream in inflammatory cascades and thus represents an appealing therapeutic target in various inflammatory conditions, including COVID-19 related cytokine storm (Favalli and Caporali, 2020). In pre-clinical studies, GM-CSF blockade reduced CAR-T-cell therapy-related toxicity by preventing cytokine release syndrome development (Sterner et al., 2019). Atypical lymphocytes expressing GM-CSF are detectable in severe COVID-19 patients (Zhou et al., 2020). Based on these observations, GM-CSF blockade was evaluated in COVID-19. Mavrilimumab is a monoclonal antibody targeting GM-CSFRα and it has been shown effective in the treatment of rheumatoid arthritis (Burmester et al., 2011). A study conducted in Milan (Italy) evaluated the efficacy of mavrilimumab in non-mechanically ventilated COVID-19 patients (De Luca et al., 2020). Specifically, 13 patients received a single intravenous dose of mavrilimumab (6 mg/kg) upon hospital admission. Outcomes at 28 days were compared to 26 patients with severe COVID-19 pneumonia and comparable baseline characteristics. Mavrilimumab was associated with a higher rate of clinical improvement (*p* = 0.03) and was well tolerated in all patients, in keeping with the good safety profile emerged in the drug development program for rheumatoid arthritis (Burmester et al., 2011). Despite clear limitations, including a small sample size and the uncontrolled nature of the investigation, this study prompted initiation of a randomized placebo-controlled trials, which is presently active in Italy (NCT04397497). Three additional monoclonal antibodies directed against GM-CSF (gimsilumab, lenzilumab, and TJ003234) are currently under investigation for the treatment of COVID-19 (NCT04351243, NCT00995449, NCT03794180).

The results of these clinical trials on GM-CSF blockade are awaited and will also address a theoretical concern related to the role of GM-CSF in the homeostasis of the alveolar surfactant. A deficit in GM-CSF has been linked to impaired differentiation of alveolar macrophages and to subsequent accumulation of surfactant components in the alveoli (Trapnell et al., 2009). Indeed, congenital deficit of GM-CSF causes the development of pulmonary proteinosis (PAP), a severe respiratory disease characterized by progressive accumulation and accumulation of exudates in the alveolar spaces. However, PAP has never been reported during the development of mavrilimumab. Similarly, PAP might not be an issue when treating COVID-19 patients, because a single intravenous dose of monoclonal antibodies typically wears off in a month, at variance with the chronic deficiency of GM-CSF observed in PAP (Bonaventura et al., 2020).

## Tumor Necrosis Factor and Janus Kinases

TNF is a mediator of paramount importance in the development of inflammatory responses. TNF levels are increased in sera of COVID-19 patients (Wang et al., 2020). It has been suggested that this cytokine is one of the very first mediators to induce tissue damage in tissues infected by coronaviruses (Haga et al., 2008).

TNF blocking agents, such as infliximab, are the cornerstone of the therapy of chronic inflammatory diseases (Smolen et al., 2020). Previous evidence suggests the potential beneficial effect of TNF inhibitors in murine models of viral pneumonia (Hussell et al., 2001). Pharmacological TNF blockade could lead to a therapeutic effect by both reducing direct inflammatory effects of this biochemical cascade and the downregulation of ACE2 expression and shedding, which are known to be essential element of viral cell entry (Haga et al., 2008). As for other biological agents, the main safety concerns for TNF inhibitors in the setting of COVID-19 patients is a raise in bacterial and fungal superinfections rates (Feldmann et al., 2020).

Retrospective data showed that infliximab was associated with clinical improvement and reduction n inflammatory markers in severe COVID-19 patients (Stallmach et al., 2020). Despite these encouraging results, no controlled evidence is available to date. Trials evaluating the role of TNF blockade in COVID-19 are currently ongoing (ChiCTR2000030089; NCT04425538—evaluating adalimumab and infliximab, respectively).

Janus kinases (JAK) are a family of mediators involved in intracellular signaling cascades downstream the receptors of multiple cytokines, most notably IL-6, but not IL-1 or TNF (O’Shea et al., 2013). Pharmacological inhibition of JAKs is an approved strategy for the treatment of various inflammatory diseases, ranging from rheumatoid arthritis and inflammatory bowel diseases to hematologic conditions (Meyer et al., 2010). In COVID-19, JAK inhibition is appealing in light of the possibility to achieve a broader modulation of inflammatory responses compared to selective blockade of individual cytokines with biologics (Seif et al., 2017; Rizk et al., 2020). Ruxolitinib is a selective inhibitor of JAK1 and JAK2 licensed for the treatment of graft-versus-host disease (Rizk et al., 2020). In a randomized clinical trial of COVID-19, treatment with ruxolitinib was associated with faster, albeit not significant, clinical improvement and a favorable safety profile (Cao et al., 2020). Ongoing trials evaluating this drug are ongoing (NCT04348071, NCT04377620, NCT04414098, NCT04362137). The JAK 1/2 inhibitor baricitinib also attracted clinical expectations, particularly following *in silico* studies postulating an inhibitory effect against viral entry into pneumocytes (Richardson et al., 2020). To date, experience with baricitinib is limited to a study evaluating combination therapy with antivirals, and reporting some degree of improvement in clinical and laboratory parameters in COVID-19 patients (Cantini et al., 2020). Ongoing trials are evaluating baricitinib or the JAK 1/3 inhibitor tofacitinib (i.e. NCT04340232, NCT04358614, NCT04345289, NCT04399798, NCT04320277). These trials will also address safety concerns related to the reported increase in thromboembolic events associated with JAK inhibitors which may further increase the hypercoagulability risk inherent to COVID-19 (Jorgensen et al., 2020; Tang Y. et al., 2020).

## Conclusions

A maladaptive, hyper-inflammatory host immune response to the virus is recognized as the main driver of disease severity in a subset of COVID-19 patients. Anti-cytokine agents with targeted anti-inflammatory effects were explored as a logical therapeutic approach in this setting. Several biotechnological drugs were repurposed for use in COVID-19, with mixed results. At present, controlled evidence indicates that IL-6 inhibition is marginally o not effective for COVID-19, whereas several uncontrolled studies evaluating IL-1 inhibition yielded overall promising results and are awaiting validation in controlled settings. Additional promising strategies include GM-CSF and JAK inhibition, although present evidence is more limited. Other theoretical options, such as TNFa inhibitor, remain relatively unexplored. Randomized clinical trials evaluating all these strategies are ongoing, but results are already available only for IL-6 inhibition. Meanwhile, as individual predisposition to the development of hyper-inflammation is revealed by COVID-19, targeted inhibition of causal cytokines is likely to confer survival benefits in some patients. Equally important, selective pharmacologic inhibition of different cytokines reveals the specific contribution of individual mediators to hyper-inflammatory responses, with translational consequences for the development of these anti-inflammatory strategies for future applications.

## Author Contributions

LD and CG conceived the manuscript. GC, NF, CC, GL, and ED-T drafted the manuscript: AT drafted the figure and table. All authors critically revised the manuscript and approved the final version.

## Funding

GC is supported by AIRC under MFAG 2018–ID. 22136, and by the FOREUM 2020 Career Grant.

## Conflict of Interest

GC received consultation honoraria from Amgen, Cerecor, Pfizer, Roche, Novartis and SOBI outside of the current work. CC received consultation honoraria from Roche and SOBI outside of the current work. GL received consultation honoraria from SOBI, Novartis, Pfizer, Celgene, Merck, and Roche outside of the current work. LD received consultation honoraria from Abbvie, Amgen, Biogen, Bristol-Myers Squibb, Celltrion, GlaxoSmithKline, Novartis, Pfizer, Roche, Sanofi-Genzyme, and SOBI outside of the current work.

The remaining authors declare that the research was conducted in the absence of any commercial or financial relationships that could be construed as a potential conflict of interest.
